# Small-molecule-based CUT&RUN for G-quadruplex DNA using a cyclic naphthalene diimide–copper complex

**DOI:** 10.1007/s44211-026-00928-8

**Published:** 2026-05-26

**Authors:** Yukina Sanada, Satoshi Fujii, Shigeori Takenaka, Shinobu Sato

**Affiliations:** 1 Department of Applied Chemistry, Kyushu Institute of Technology, 1-1 Sensui-cho, Tobata-ku, Kitakyushu-shi, Fukuoka 804-8550 Japan; 2 Department of Bioscience and Bioinformatics, Kyushu Institute of Technology, 680-4, Kawazu, Iizuka-shi, Fukuoka 820-8502 Japan

**Keywords:** G-quadruplex DNA (G4), cyclicnaphthalene diimide, G4 DNA cleavage, G4-targeted chromatin analysis, CUT&RUN, Cu–ATCUN motif

## Abstract

G-quadruplex (G4) DNA structures are widely distributed in biologically important genomic regions and are involved in gene regulation and genome stability. However, experimental identification of G4 sites in chromatin remains challenging. Here, we report a multifunctional small-molecule ligand, cNDI-CuGGHE, that integrates selective G4 recognition and copper-mediated DNA cleavage within a single molecular scaffold. Spectroscopic analysis showed that cNDI-CuGGHE preferentially binds to various G4 structures through terminal stacking on the G-quartet plane with binding constants on the order of 10⁶ M⁻¹ and significantly stabilizes G4 DNA compared with duplex DNA. Gel electrophoresis confirmed efficient and structure-dependent DNA cleavage in the presence of sodium ascorbate and hydrogen peroxide. Genomic DNA recovered from a cNDI-CuGGHE-based CUT&RUN-like assay in HeLa cells was analyzed by quantitative PCR using primers flanking the G4-forming regions of the c*-kit* and c-*myc* promoters. The reduced amplification of G4-spanning regions was consistent with preferential DNA cleavage at G4 sites in chromatin. These results demonstrate the feasibility of using small-molecule ligands as alternatives to antibody–enzyme conjugates for G4-targeted chromatin analysis.

**Graphical abstract:**

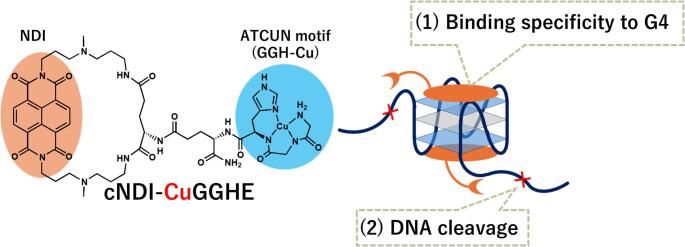

**Supplementary Information:**

The online version contains supplementary material available at 10.1007/s44211-026-00928-8.

## Introduction

Guanine-rich DNA sequences can form G-quadruplex (G4) structures through the stacking of G-quartets stabilized by monovalent cations such as K⁺ and Na⁺ [1]. These non-canonical DNA structures are widely distributed in the human genome and are particularly enriched in biologically important regions, including telomeres, gene promoters, and replication origins [2]. Accumulating evidence indicates that G4 structures play important roles in the regulation of gene transcription, DNA replication, and genome stability [3, 4]. Recent comprehensive studies have further highlighted the broader analytical and biological significance of higher-order nucleic acid structures, including G4 DNA, in cellular regulation and disease contexts [5]. In particular, the enrichment of G4-forming sequences in oncogene promoters and telomeric regions has attracted considerable attention, because G4 formation is closely related to tumorigenesis and cellular proliferation [3, 4]. Because the formation and stability of G4 structures are highly dependent on the chromatin environment and cellular conditions, the precise identification of G4 sites in living cells remains a major analytical challenge. Although bioinformatic prediction can identify potential G4-forming sequences, these approaches cannot determine whether G4 structures are formed under physiological conditions. Therefore, experimental methods that can directly detect G4 structures in genomic DNA are essential for understanding their biological functions.

Several experimental and analytical strategies have been developed to detect and characterize G4 structures in cells, reflecting increasing recognition of their functional and biomedical importance [6]. Antibody-based approaches, including BG4-mediated chromatin immunoprecipitation followed by sequencing (ChIP-seq), have been widely used to profile G4 structures genome-wide [7, 8]. More recently, Cleavage Under Targets and Release Using Nuclease (CUT&RUN) has emerged as a powerful alternative to conventional ChIP-based methods [9]. In CUT&RUN, an antibody–nuclease conjugate selectively binds to a target molecule and cleaves genomic DNA in its vicinity, allowing highly specific recovery of DNA fragments under mild conditions with low background and improved resolution.

While these antibody-based strategies have significantly advanced G4 mapping, they rely on protein reagents and require careful optimization to minimize potential perturbation of chromatin environments. In addition, the relatively large size of antibodies may limit accessibility to compact chromatin regions and sterically hinder binding to certain DNA structures. These considerations motivate the development of alternative G4 detection strategies based on small-molecule ligands, which generally exhibit higher accessibility, improved chemical tunability, and enhanced reproducibility.

Cyclic naphthalene diimide (cNDI) derivatives are well-established G4 ligands that selectively bind G-quartet planes and represent an important class of chemically programmable G4-recognition molecules [10–13]. We have previously shown that biotinylated cNDI can be used as a G4-selective probe to recover G4-containing genomic DNA fragments, demonstrating its potential as an analytical tool for G4 research [11]. Building on this concept, integration of a DNA-cleaving moiety into a G4-binding ligand offers a promising strategy to develop a small-molecule-based alternative to antibody–enzyme conjugates used in CUT&RUN.

**Fig. 1:**
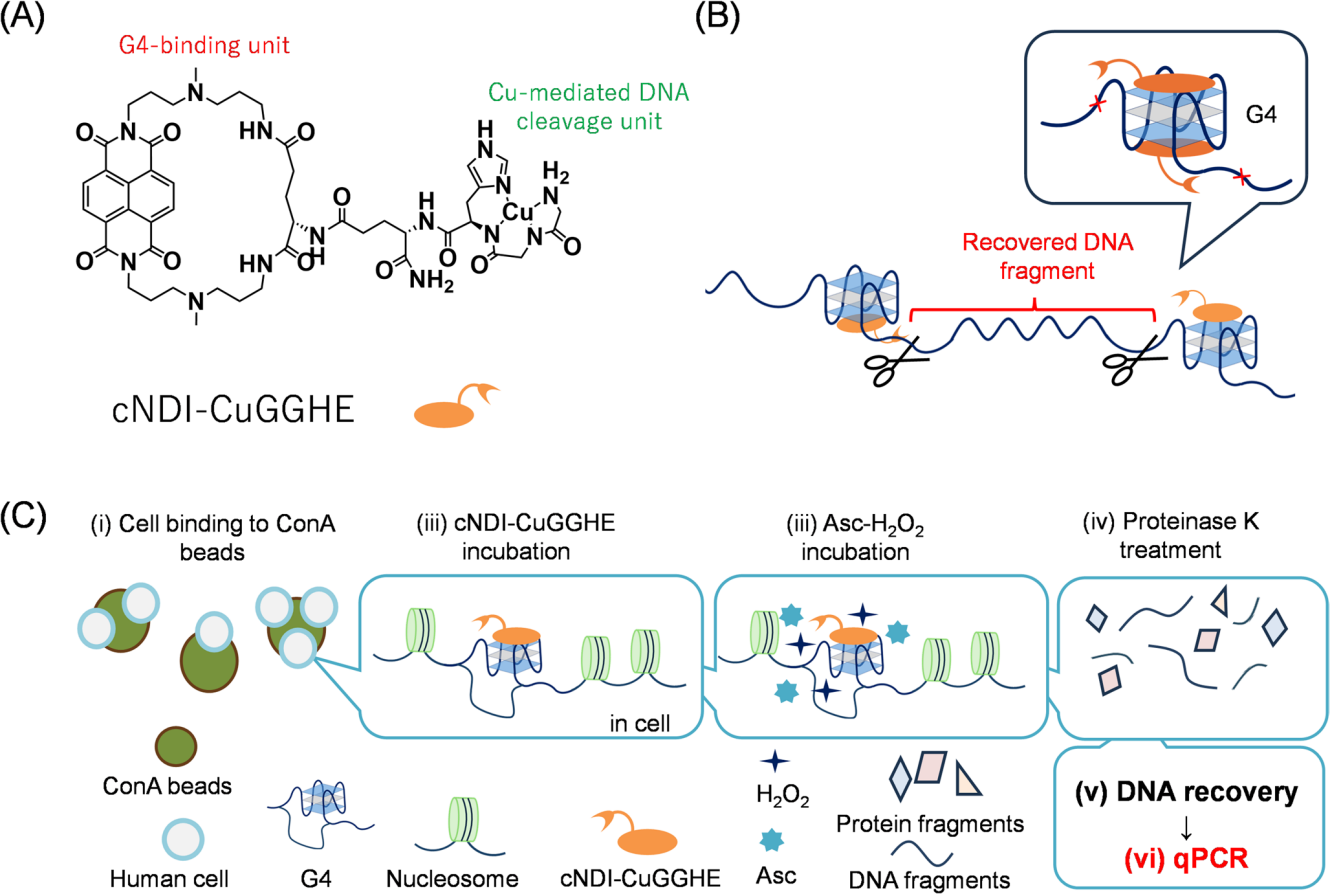
Schematic illustration of the small-molecule-based CUT&RUN-like strategy using cNDI-CuGGHE for selective recognition, chemical cleavage, and recovery of G4-associated genomic DNA. **A** Chemical Structure of cNDI-CuGGHE, **B** Image of cNDI-CuGGHE binding to G-Quadruplex DNA (G4) on the genome and cleaving DNA, **C** Strategy of cNDI-CuGGHE-Based CUT&RUN for G4

In this study, we designed and synthesized a novel cNDI derivative bearing an ATCUN (amino-terminal Cu²⁺ and Ni²⁺-binding) motif [14], cNDI-CuGGHE (Fig. 1A), which combines G4 recognition and copper-mediated DNA cleavage within a single small molecule. Upon binding to a G4 structure, the cNDI moiety anchors the compound on the G-quartet plane, while the Cu–ATCUN motif generates reactive oxygen species in the presence of sodium ascorbate and hydrogen peroxide, leading to preferential DNA cleavage near the G4 site (Fig. 1B). We hypothesized that this design would enable selective cleavage at G4 sites in chromatin, thereby allowing recovery and analysis of G4-associated genomic DNA fragments (Fig. 1C).

Here, we report the molecular design, in vitro characterization, and cellular application of cNDI-CuGGHE as a small-molecule-based G4 detection tool. The interaction of cNDI-CuGGHE with various G4 and non-G4 DNA structures was evaluated by UV–visible absorption spectroscopy, circular dichroism spectroscopy, melting temperature analysis, and polyacrylamide gel electrophoresis. Furthermore, we applied cNDI-CuGGHE to a CUT&RUN-like assay in human cells, followed by quantitative PCR to demonstrate selective DNA cleavage at G4-forming genomic regions. This work establishes a small-molecule-based analytical platform for G4 mapping and provides a versatile framework for studying G4 structures in living cells.

## Experimental

### Materials

The synthetic oligonucleotides listed in Table 1 were custom synthesized by Hokkaido System Science (Hokkaido, Japan). The concentrations of the oligonucleotide and 71 bp DNA in this experiment are shown as concentrations per strand. Sodium ascorbate (NaAsc, Tokyo Chemical Industry Co., Ltd., Tokyo, Japan), hydrogen peroxide (H₂O₂, FUJIFILM Wako Pure Chemical Co., Tokyo, Japan), CuCl₂, and other reagents were of analytical grade and used without further purification. Ultrapure water was obtained from a Milli-Q system. cNDI-GGHE was synthesized from Fmoc-Glu(cNDI)-OH [12]. The synthesis details are shown in Supplementary Information, and ^1^H-NMR cNDI-NMe-Glu-NH_2_ and the HPLC and ESI-MS data for cNDI-GGHE are shown in Figs. S1 and S2, respectively. The purity was confirmed by analytical RP-HPLC, and identity was confirmed by ESI-MS.

**Table 1:** Oligonucleotides used in these experiments

DNAs	Sequence (5′ to 3′)
c-*myc*	TGAGGGTGGGTAGGGTGGGTAA
c-*kit*	AGGGAGGGCGCTGGGAGGAGGG
TA-core	TAGGGTTAGGGTTAGGGTTAGGG
G2T1	AGGGTTAGGGTTAGGGTTAGGGTTAGGGTTAGGGTTAGGGTTAGGG
12-ss (-) 12-ss (+)	GCGAAACCTCCC GGGAGGTTTCGC
HP-27^*^	*CGCGAATTCGCG*TTT*CGCGAATTCGCG*
duplex DNA 71^**^	GGTAGTTGGAGCTGTTGGCGTAGGCAAGAGTGCCTTGACGATACAGCTAATTCAGAATCATTTTGTGGACG
F-Primer	GGTAGTTGGAGCTGTTGGC
R-Primer	CGTCCACAAAATGATTCTGAATTAGCTGT

^*^The italics part can form a double strand

^**^The sequence shown is only a portion of the plus strand of the K-RAS gene. PCR was performed using F-primer and R-primer

## Preparation of G4 DNA and cNDI-CuGGHE

DNA samples were dissolved in 50 mM AcOH–AcOK buffer (pH 5.5) containing 100 mM KCl, heated at 95 °C for 5 min, and slowly cooled to room temperature (− 1 °C/min). Annealed DNA solutions were stored at 4 °C and diluted to the desired concentrations immediately before use. cNDI-GGHE (1000 µM) was dissolved in water to prepare a stock solution. cNDI-CuGGHE was prepared by mixing cNDI-GGHE with an equimolar amount of 1000 µM of CuCl₂ in buffer, followed by gentle mixing at room temperature for 5 min. The resulting solution was used immediately for spectroscopic and cleavage experiments. For control experiments, cNDI-GGHE without copper or CuCl₂ alone was prepared under identical conditions.

## Absorption titration of cNDI-CuGGHE or cNDI-GGHE with DNAs

UV–visible absorption spectra were recorded at 25 °C using a quartz cuvette with a 1 cm path length with JASCO V-750 Spectrophotometer (Tokyo, Japan). Titration experiments were performed by incremental addition of DNA to a solution of cNDI-CuGGHE (6.0 µM) in 50 mM AcOH–AcOK buffer (pH 5.5) containing 100 mM KCl. The binding constant (*K*ₐ) and binding stoichiometry (n) were determined by plotting the absorbance ratio (1-*A*/*A*₀) against the total DNA concentration (*S*_T_) and fitting the absorbance change at 384 nm using the binding isotherm model [15] (Eq. (1)).

1 $$\:1-\frac{A}{{A}_{0}}=\frac{1}{2}{R}_{ｂ}\left\{\frac{1}{{Ｋ}_{a}}+{L}_{T}+n{S}_{T}-\sqrt{{\left(\frac{1}{{K}_{a}}+{L}_{T}+n{S}_{T}\right)}^{2}-4n{S}_{T}{L}_{T}\:}\right\}$$

where *A*: absorbance, *A*_*0*_: initial absorbance ([DNA] = 0 M), *K*_a_: binding constant, *L*_T_: total ligand concentration *S*_T_: total DNA concentration, n: number of bindings, and *R*_b_: instrument response sensitivity.

## Circular dichroism (CD) measurements

CD spectra were recorded at 25 °C using a 1 mm path-length quartz cell with JASCO J-820 circular dichroism spectrometer. DNA samples (typically 1.5 µM) in 50 mM AcOH-AcOK buffer (pH 5.5) and 100 mM KCl were measured in the absence and presence of cNDI-CuGGHE. Thermal melting experiments were performed by monitoring the CD signal at the characteristic wavelength for G4 structures while increasing the temperature at a constant rate.

## Tm measurements

Tm was measured in 1.5 µM DNA in 50 mM AcOH-AcOK buffer (pH 5.5) and 100 mM KCl before and after adding ligand. CD absorption changes at 263 nm for c-*myc* and c-*kit* or 288 nm for TA-core and G2T1 were measured for G4 DNA ; Temperature gradient: 1 °C/min (20–95 °C).

For ds-oligo as double-stranded DNA, the temperature dependence of the change in absorption intensity at 260 nm was measured with JASCO V-750 Spectrophotometer (Temperature gradient: 1 °C/min).

### DNA cleavage assay using polyacrylamide gel electrophoresis (PAGE)

A 71-bp double-stranded DNA was used as the long double stranded DNA. PCR was performed using 50 ng/µL duplex DNA 71 as the template, 0.3 µM F-Primer, 0.3 µM R-Primer, and 1x HotStarTaq Master Mix (QIAGEN) (95 °C for 5 min × 1, (95 °C, 15 s, 63 °C for 30 s, 72 °C for 30 s) ). The resulting PCR product was purified using a Nucleo Spin Gel and PCR Clean-up Kit (Takara Bio, Tokyo, Japan).

DNA cleavage reactions were carried out by incubating DNA (typically 1.5 µM, 71-bp duplex DNA for 0.0063 µM) with cNDI-CuGGHE (typically 10 µM, 0.417 µM for 71-bp duplex DNA) in the presence of 1.0 mM NaAsc and 1.0 mM H₂O₂ at 37 °C. Reactions were quenched by the addition of EDTA and analyzed by denaturing 15% polyacrylamide gel electrophoresis (PAGE). Control experiments were performed in the absence of cNDI-CuGGHE, NaAsc, or H₂O₂, or using cNDI-GGHE without copper. Cleavage products were also analyzed by reversed-phase HPLC using a C18 column (Mightysil RP-18). Elution profiles were monitored by UV absorbance, and retention times of cleavage fragments were compared with those of intact DNA.

## Cell-based CUT&RUN-like assay [16]

Ten µL of 5 mg/mL concanavalin A-coated magnetic beads (BioMagPlus Concanavalin A, Bangs Laboratories, Inc.) were washed with 1 mL of binding buffer (20 mM HEPES (pH 7.5), 10 mM KCl, 1 mM CaCl_2_, 1 mM MnCl_2_) using a magnetic stand (×2), and 50 µL of binding buffer was added to the beads. Next, 400 µL of a HeLa cell suspension (2.5 × 10⁵ cells/mL, JCRB9004, LotNo. 10252012) in PBS was added to each tube, gently mixed by tapping, and allowed to stand at room temperature for 10 min. The beads were then captured using a magnetic stand, and the supernatant was removed. 100 µL of a 30 µM cNDI-CuGGHE solution prepared in Dig Wash Buffer (20 mM HEPES (pH7.5), 150 mM NaCl, 0.5 mM Spermidine trihydrochloride, 0.02% Digitonin containing cOmplete Mini, EDTA-free) was added. These were incubated overnight at 4 °C and 500 rpm. The next day, the supernatant was removed using the magnetic stand, and 90 µL of NaAsc and 90 µL of H₂O₂ were added to each tube. The tubes were mixed for 10 s at 500 rpm and then allowed to stand at 4 °C for 20 min. To terminate the reaction, 20 µL of 10× stop buffer (1700 mM NaCl, 100 mM EDTA, 20 mM EGTA, 0.02% Digitonin, 250 µg/mL RNase A, 500 µg/mL Glycogen) was added to each tube, mixed at 500 rpm, and incubated at 37 °C and 750 rpm for 20 min. The resulting supernatant was transferred to a new tube, and 2 µL of 10% SDS solution and 2.5 µL of proteinase K (20 mg/mL) were added. The mixture was then incubated at 50 °C for 60 min. After 2 µL of carrier RNA (10 ng/µL) was added, DNA was extracted with 200 µL of phenol: chloroform: isoamyl alcohol (25:24:1), precipitated with 20 µL of 3 M sodium acetate, 2 µL of glycogen (20 mg/mL), and 750 µL of 99.5% ethanol, and then incubated overnight at − 20 °C.

## qPCR verification

Pull down DNA was used to quantify via qPCR with a Stratagene Mx3005P (Agilent Technologies) and QuantiTect SYBR Green PCR *Kit* (QIAGEN). Reaction mixture (50 µL) contained 1 × QuantiTect SYBR Green PCR Master Mix, 0.3 µM FP, 0.3 µM RP, and 1 µL pulldown DNA. Cycling conditions were 95 °C for 25 min, 45 × (95 °C for 15s, 60 °C for 30s, 72 °C for 30s). FP and RP were prepared for the c-*myc* and c-*kit* regions, respectively, in region 1 and region 2. Region 3 was the region amplified by FP1 in Region 1 and RP2 in Region 2. qPCR primers are listed in Table S1. Each experiment was performed independently in triplicate. Ct values are reported as mean ± SD. Samples showing no amplification within 45 cycles were treated as non-detected. Relative DNA abundance was evaluated using ΔCt and 2^⁻ΔCt^ calculations.

## Results

### Interaction Analysis of ligand with several DNAs

**Fig. 2:**
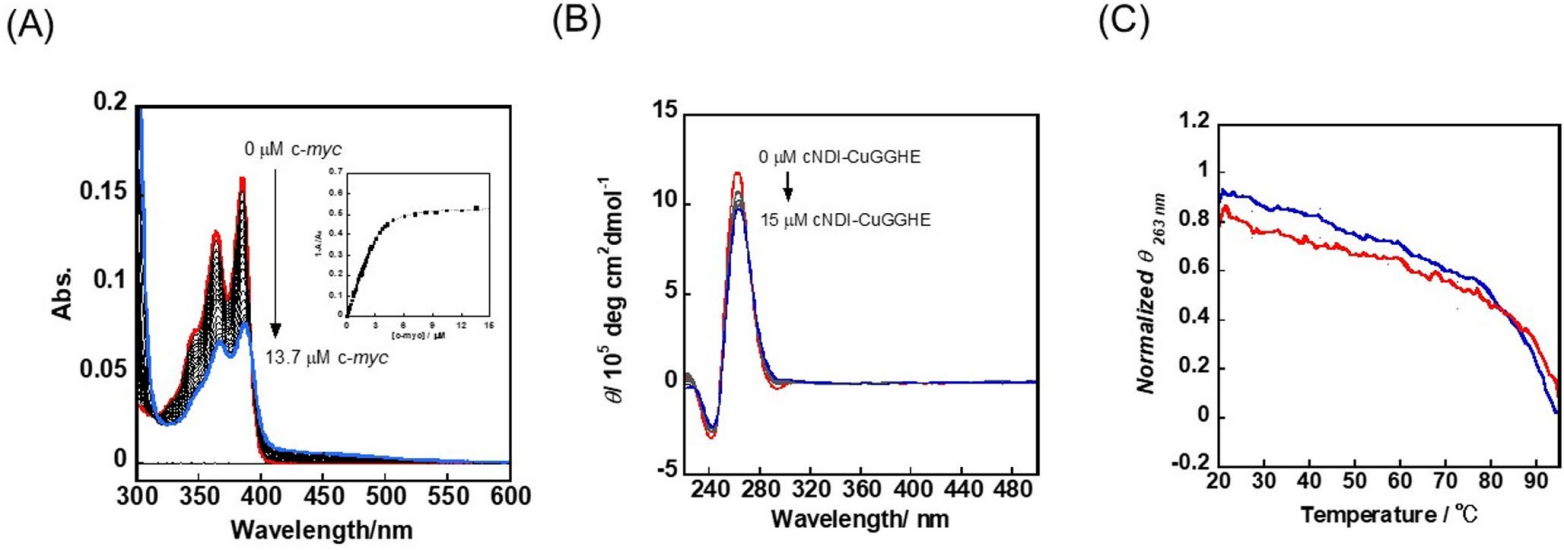
Interaction of cNDI-CuGGHE with c-*myc*. **A** Absorption titration of 6.0 µM cNDI-CuGGHE with addition of c-*myc*, **B** CD spectra of 1.5 µM c-*myc* upon addition of cNDI-CuGGHE (0, 1.5, 3, 6, 10.5 or 15 µM from upper to bottom), and **C** Tm measurement of 1.5 mM c-*myc* in the absence or presence of cNDI-CuGGHE (1:2 molar ratio) in 50 mM AcOH-AcOK (pH 5.5), 100 mM KCl

The interaction of cNDI-CuGGHE with DNA was first evaluated by UV–visible absorption spectroscopy. cNDI-CuGGHE exhibited a characteristic absorption band at approximately 384 nm arising from the π–π* transition of the naphthalene diimide chromophore. When c-*myc* G4 DNA was added to a solution of 6 µM cNDI-CuGGHE in 50 mM AcOH–AcOK buffer (pH 5.5) containing 100 mM KCl, a pronounced hypochromic effect accompanied by a slight red shift was observed (Fig. 2A). Such spectral changes are typically observed for π–π stacking interactions between planar aromatic ligands and the G-quartet planes of G4 structures [10], indicating that cNDI-CuGGHE binds to the terminal G-quartets of G4 DNA by end-stacking. Similar spectral changes were observed upon addition of other G4 DNAs, including c-*kit*, TA-core, and G2T1 (Fig. S3). In contrast, the addition of double-stranded DNA (ds-oligo) or single-stranded DNA (12-ss) induced little change in the absorption spectrum (Fig. S3), demonstrating the high selectivity of cNDI-CuGGHE for G4 structures over canonical DNA forms.

The changes in absorbance at 384 nm were analyzed using a binding isotherm model, and the resulting binding constants, binding stoichiometries, and hypochromicity ratios are summarized in Table 2. The binding constants for G4 DNAs were on the order of 10⁶ M⁻¹, which were at least two orders of magnitude higher than those for dsDNA and ssDNA. These results indicate that the introduction of the Cu–ATCUN motif does not compromise the intrinsic G4 selectivity of the cNDI scaffold. Similar UV–vis titration experiments were also performed using cNDI-GGHE without copper (Fig. S4 and Table 2), yielding nearly identical hypochromic responses and binding constants, indicating that copper coordination has little effect on G4 binding behavior. In addition, the hypochromicity at 384 nm reached 52–59% for G4 DNAs, which is characteristic of strong π–π stacking of naphthalene diimide derivatives on G-quartet planes, whereas dsDNA and ssDNA showed much smaller hypochromicity of 37–41% and 16–18%, respectively (Table 2). The fitting model assumed independent binding sites with experimentally derived stoichiometry values, consistent with end-stacking at terminal G-quartets. The binding stoichiometry (n) was approximately 2 per G4 unit, and G2T1, which contains two G4 units, accommodated four molecules of cNDI-CuGGHE. This stoichiometry is fully consistent with an end-stacking binding mode on the terminal G-quartets.

**Table 2:** Binding parameters of cNDI-CuGGHE or cNDI-GGHE with the several DNAs

Compound	DNA	*K*_a_/10^6^M^− 1^	*n*	hyperchromicity/%
cNDI-CuGGHE	c-*myc*	1.7	2	53
	c-*kit*	0.36	2	52
	TA-core	1.5	2	59
	G2T1	4.7	4	59
	ds-oligo	0.13	1	37
	12-ss (-)	0.014	-	16
cNDI-GGHE	c-*myc*	2.3	2	53
	c-*kit*	0.50	2	53
	TA-core	3.1	2	60
	G2T1	3.6	4	59
	ds-oligo	0.35	1	41
	12-ss (-)	0.098	-	18

The effect of ligand binding on the G4 conformation was then examined by circular dichroism (CD) spectroscopy. The CD spectrum of c-*myc* DNA (Fig. 2B) exhibited a positive Cotton effect at approximately 260 nm and a negative band at around 240 nm, which are characteristic of a parallel G4 topology. Similar spectral features were observed for c-*myc* and c-*kit*, whereas TA-core and G2T1 showed CD signatures corresponding to hybrid-type G4 structures (Fig. S5). Importantly, addition of cNDI-CuGGHE or cNDI-GGHE caused no significant changes in the CD spectral profiles of any of the G4 DNAs, indicating that ligand binding does not perturb the native G4 topology. This is consistent with end-stacking on the G-quartet plane without affecting loop conformations or strand orientations.

Taken together, the UV–Vis and CD data demonstrate that cNDI-CuGGHE retains the characteristic binding mode of cNDI derivatives, namely selective end-stacking onto G-quartet planes, while preserving the structural integrity of the G4 fold. This behavior is particularly important for CUT&RUN-type applications, as it ensures that DNA cleavage is triggered at pre-existing G4 structures rather than being induced by ligand-driven conformational changes. Therefore, cNDI-CuGGHE functions as a G4-selective molecular anchor that positions the Cu–ATCUN catalytic center in close proximity to native G4 sites.

The stabilizing effect of cNDI-CuGGHE or cNDI-GGHE on G4 DNA was evaluated by monitoring the temperature dependence of circular dichroism (CD) signals, and melting temperatures (Tm) were determined in the absence and presence of the ligand (Figs. 2C and S6). In the absence of cNDI-CuGGHE, all G4 DNAs exhibited characteristic thermal melting profiles corresponding to dissociation of G-quartet stacking. Upon addition of cNDI-CuGGHE at a 2-fold molar excess relative to DNA, a clear increase in Tm was observed for all G4 sequences examined.

For the parallel-type G4 structure c-*myc*, the presence of cNDI-CuGGHE resulted in a pronounced elevation of Tm, indicating strong stabilization of the folded G4 structure (Fig. 2C). Similarly, hybrid-type G4s such as TA-core and G2T1 exhibited significant increases in Tm by 4–15 °C upon ligand binding (Table 2, Fig. S6), c-*kit* G4 also showed significant thermal stabilization upon binding of cNDI-CuGGHE under identical conditions, further supporting internal consistency between in vitro thermodynamic characterization and cellular qPCR analysis. In contrast, the melting temperature of dsDNA was essentially unchanged in the presence of cNDI-CuGGHE, demonstrating that the stabilization effect is highly selective for G4 structures.

As summarized in Table 3, similar stabilization was also observed for cNDI-GGHE without copper, indicating that the cNDI moiety is primarily responsible for G4 stabilization. These results show that cNDI-CuGGHE not only binds to G4 DNA but also stabilizes the G4 fold, which is consistent with end-stacking of the cNDI unit on the terminal G-quartet planes. Importantly, this stabilization occurs without inducing any detectable change in G4 topology, as demonstrated by the CD measurements, indicating that the ligand reinforces pre-existing G4 structures rather than driving conformational rearrangements.

**Table 3:** Thermal stability enhancement of cNDI-CuGGHE or cNDI-GGHE for DNA structure

Compound	DNA	*n*	T_m_ (0 eq)/°C	T_m_ (*n* eq.)/°C	ΔT_m_/°C
cNDI-CuGGHE	c-*myc*	2	90	> 95	> 5
	TA-core	2 2	68 69	72 75	4 6
	c-*kit*				
	G2T1	4	60	71	11
	ds-oligo	1	52	52.5	0.5
cNDI-GGHE	c-*myc*	2	90	> 95	> 5
	TA-core	2 2	68 69	72.5 78.5	4.5 9.5
	c-*kit*				
	G2T1	4	60	75	15
	ds-oligo	1	52	52	0

The selective stabilization of G4 structures by cNDI-CuGGHE is particularly important for its application in CUT&RUN-type assays. Stabilization of G4 DNA is expected to prolong the lifetime of G4 conformations in chromatin and to increase the residence time of the ligand at G4 sites, thereby enhancing the probability of preferential DNA cleavage by the Cu–ATCUN catalytic center. Therefore, the thermal stabilization observed here supports the suitability of cNDI-CuGGHE as a molecular anchor for G4-targeted genome analysis.

### G4-selective DNA cleavage by cNDI-CuGGHE

**Fig. 3:**
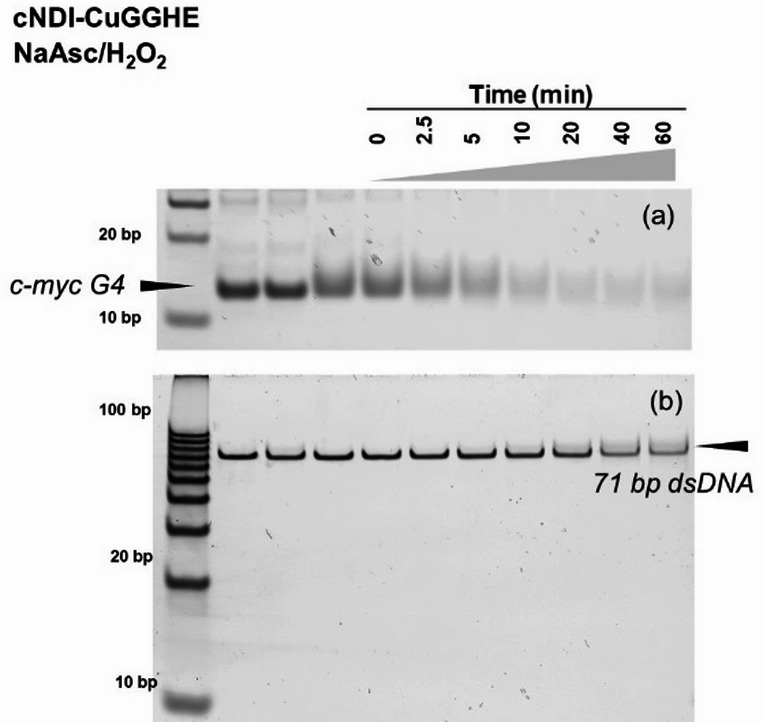
Gel electropherogram for DNA cleavage by cNDI-CuGGHE. c-*myc* (**a**) or 71 bp dsDNA (**b**) was treated with 1.0 mM NaAsc and 1.0 mM H_2_O_2_ at 37 °C

The DNA cleavage activity of cNDI-CuGGHE was evaluated by polyacrylamide gel electrophoresis (PAGE) and reversed-phase HPLC in the presence of NaAsc and H₂O₂. As shown in Fig. 3a, c-*myc* DNA alone exhibited a single band corresponding to approximately 15 bp. Upon incubation with cNDI-CuGGHE, the appearance of lower-molecular-weight bands and smearing was observed, indicating DNA cleavage. Because no discrete cleavage fragments were detected, the DNA was likely degraded to mono- or dinucleotide-sized products. Similar cleavage behavior was observed for other G4-forming DNAs, including c-*kit*, TA-core, and G2T1 (Fig. S7) and cleavage was time-dependent.

In contrast, under the same conditions, ds-oligo and a hairpin DNA containing a 12-bp duplex stem (HP27) showed only limited cleavage compared with G4 DNAs (Fig. S7). The ds-oligo is known to bind cNDI-CuGGHE at its termini (Table 2), which likely accounts for the observed weak cleavage. Consistently, the hairpin DNA HP27, which possesses only a single duplex terminus, was less cleaved than the duplex oligonucleotide, whereas a 71-bp duplex DNA fragment derived from the gene showed almost no degradation (Fig. 3b). These results indicate that DNA cleavage by cNDI-CuGGHE occurs preferentially in the presence of G4 structures and is minimal for long duplex DNA.

Control experiments confirmed that all components of the catalytic system are required for DNA cleavage. In the absence of cNDI-CuGGHE, or when either NaAsc or H₂O₂ was omitted, no significant DNA degradation was observed. Moreover, cNDI derivatives lacking the Cu-binding GGHE motif exhibited no detectable cleavage activity, demonstrating that the metal-binding site is essential for catalysis.

HPLC analysis confirmed the PAGE results. Incubation of G4 DNAs, c-*myc* and TA-core, with cNDI-CuGGHE, NaAsc and H_2_O_2_ produced multiple cleavage products that eluted with shorter retention times (RT, 3–4 min) than intact DNA (Fig. S8). MS analysis of these fractions showed peaks at m/z 320–340 (data not shown), suggesting that DNA cleavage may have occurred down to the mononucleotide level. This behavior is consistent with a proximity-driven mechanism in which the cNDI moiety anchors the Cu-ATCUN catalytic center in a G4 structure, locally generating reactive oxygen species and enabling site-selective DNA cleavage.

### qPCR verification of G4-selective DNA cleavage in cells

The ability of cNDI-CuGGHE to induce DNA cleavage at G4 sites in chromatin was evaluated using a CUT&RUN-like assay in HeLa cells, followed by quantitative PCR (qPCR) analysis of the recovered DNA fragments. After permeabilization, cells were incubated with cNDI-CuGGHE and activated by sodium ascorbate and hydrogen peroxide to trigger preferential DNA cleavage. The released DNA fragments were purified and analyzed by qPCR using primer sets designed to interrogate G4-forming regions in the c-*kit* and c-*myc* promoters (Fig. S9).

**Fig. 4:**
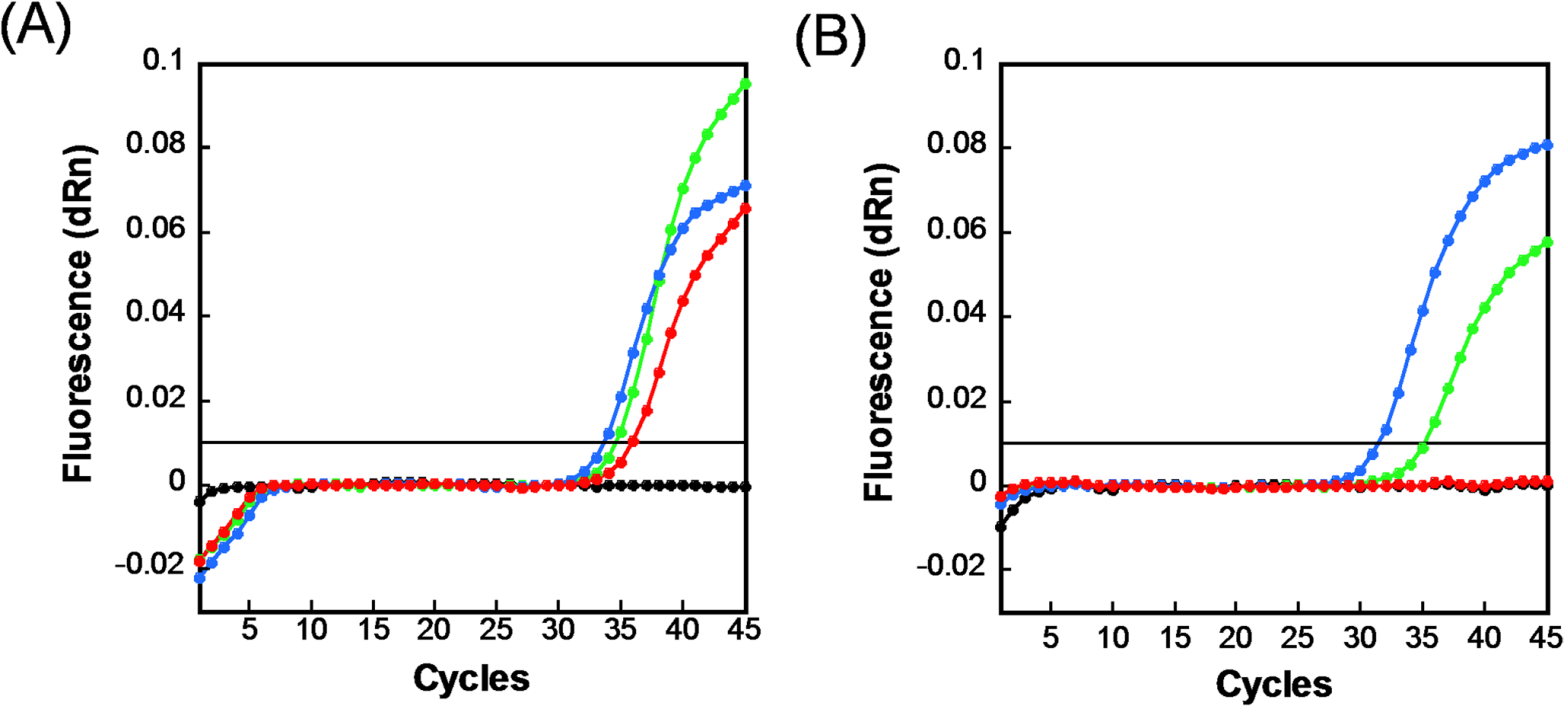
qPCR after pulldown of genome DNA fragments by cNDI-CuGGHE based CUT &RUN. (A) qPCR (green; FP1/RP1, blue; FP2/RP2, red; FP1/RP2, black; no genome with FP1/RP2) carried out with c-*kit* Primer set (**A**) or c-*myc* Primer set (**B**)

For the c-*kit* locus (Fig. 4A), Ct values (mean ± SD) were 34.33 ± 0.76 for FP1/RP1 (*n* = 3), 33.50 ± 0.00 for FP2/RP2 (*n* = 3), and 35.40 ± 0.85 for FP1/RP2 (*n* = 3). Using the average Ct of the flanking amplicons as a reference (Ct = 33.92), the FP1/RP2 amplicon spanning the G4 region showed a ΔCt of 1.48, corresponding to a 2^−ΔCt^ value of 0.36 (2.8-fold lower), indicating preferential loss of amplifiable DNA across the G4 site.

For the c-*myc* locus (Fig. 4B), the flanking amplicons yielded Ct values of 34.60 ± 0.35 (FP1/RP1, *n* = 3) and 31.50 ± 0.10 (FP2/RP2, *n* = 3), whereas no amplification of the FP1/RP2 amplicon was detected within 45 cycles (Ct not determined), demonstrating near-complete loss of DNA continuity across the G4-forming region.

Importantly, in control experiments performed without cNDI-CuGGHE or without oxidative activation, the FP1/RP2 amplicons for both c-*kit* and c-*myc* were readily detected within 45 cycles, confirming that the G4-spanning regions are intrinsically amplifiable and that the observed signal loss is dependent on cNDI-CuGGHE-mediated cleavage.

Notably, the more pronounced loss of amplifiable DNA at the c-*myc* locus compared with c-*kit* is fully consistent with the approximately fourfold higher binding affinity of cNDI-CuGGHE for the c-*myc* G4, as determined by UV–visible titration (Table 2). This correlation supports an affinity-driven proximity cleavage mechanism in which stronger G4 binding results in more efficient localization of the Cu–ATCUN catalytic center and, consequently, enhanced DNA cleavage at the target site.

Together, these qPCR results provide quantitative and locus-specific evidence that cNDI-CuGGHE functions as a small-molecule CUT&RUN reagent capable of selectively interrogating G4 structures in cellular chromatin. While mechanistically distinct from conventional enzyme-tethered CUT&RUN, this chemically induced cleavage strategy is conceptually analogous in enabling selective recovery of DNA fragments adjacent to target structures.

## Conclusions

We developed a multifunctional small-molecule ligand, cNDI-CuGGHE, that combines selective G-quadruplex (G4) recognition with copper-mediated DNA cleavage. The ligand binds to G4 structures through end-stacking on the G-quartet planes without altering their native topologies and selectively stabilizes G4 DNA over duplex DNA. In the presence of sodium ascorbate and hydrogen peroxide, cNDI-CuGGHE induces structure-dependent DNA cleavage at G4 sites.

Application of cNDI-CuGGHE to a CUT&RUN-like assay in human cells enabled selective interrogation of G4-forming regions, as verified by quantitative PCR. These results demonstrate the feasibility of using small-molecule ligands as alternatives to antibody–enzyme conjugates for G4-targeted chromatin analysis and provide a basis for further development of sequencing-based G4 mapping approaches. This strategy may provide a practical platform for future sequencing-based G4 mapping and potentially for broader chromatin structure analysis using chemically programmable small molecules.

## Electronic Supplementary Material

Below is the link to the electronic supplementary material.

Supplementary Material 1

## Data Availability

The data supporting the findings of this study are available within the paper and its Supplementary Information files. Should any raw data files be needed in another format they are available from the corresponding author upon reasonable request.

## References

[1] G. Sen, W. Gilbert, Nature (1988). 10.1038/334364a0 10.1038/334364a0 3393228

[2] J.L. Huppert, S. Balasubramanian, Nucleic Acids Res. (2005). 10.1093/nar/gki609 15914667 10.1093/nar/gki609 PMC1140081

[3] R. Siddiqui-Jain, C.L. Grand, D.J. Bearss, L.H. Hurley, Proc. Natl. Acad. Sci. U.S.A. (2002). 10.1073/pnas.182256799 12195017 10.1073/pnas.182256799 PMC129314

[4] S. Neidle, Nat. Rev. Chem. (2017). 10.1038/s41570-017-0041

[5] J. Wu, Y. Li, L. Wang, S.-C. Nao, D.S.-H. Chan, C.-Y. Wong, G. Yang, W. Wang, C.-H. Leung, TrAC Trends Anal. Chem. (2025). 10.1016/j.trac.2024.118121

[6] B. Roy, T. Govindaraju, Bull. Chem. Soc. Jpn. (2024). 10.1093/bulcsj/bcsj.20230224

[7] G. Biffi, D. Tannahill, J. McCafferty, S. Balasubramanian, Nat. Chem. (2013). 10.1038/nchem.1548 23422559 10.1038/nchem.1548 PMC3622242

[8] V.S. Chambers, G. Marsico, J.M. Boutell, M.D. Antonio, G.P. Smith, S. Balasubramanian, Nat. Biotechnol. (2015). 10.1038/nbt.3295 26192317 10.1038/nbt.3295

[9] P.J. Skene, S. Henikoff, eLife (2017). 10.7554/eLife.21856 10.7554/eLife.21856 PMC5310842 28079019

[10] Y. Esaki, M.M. Islam, S. Fujii, S. Sato, S. Takenaka, Chem. Commun. (2012). 10.1039/C4CC01005A 10.1039/c4cc01005a 24752309

[11] S. Fujii, S. Sato, R. Hidaka, S. Takenaka, Anal. Sci. (2024). 10.1007/s44211-024-00551-5 38609708 10.1007/s44211-024-00551-5 PMC11035424

[12] K. Ono, S. Sato, S. Takenaka, ChemistrySelect (2024). 10.1002/slct.202401062

[13] L. D’Anna, L. Marretta, A. Froux, S. Rubino, V. Butera, A. Spinello, R. Bonsignore, A. Terenzi, G. Barone, Eur. J. Inorg. Chem. (2025). 10.1002/ejic.202400705

[14] Y. Jin, J.A. Cowan, J. Am. Chem. Soc. (2005). 10.1021/ja0503985 15941274 10.1021/ja0503985

[15] F.H. Stootman, D.M. Fisher, A. Rodgerc, J.R. Aldrich-Wright, Analyst (2006). 10.1039/B604686J 10.1039/b604686j 17003863

[16] A. Inoue, Z. Chen, Q. Yin, Y. Zhang, Genes Dev. (2018). 10.1101/gad.318675.118 30463900 10.1101/gad.318675.118 PMC6295166

