# Supplementary material for: Small-molecule-based CUT&RUN for G-quadruplex DNA using a cyclic naphthalene diimide–copper complex
Source: Anal Sci. 2026 May 26;42(8):759–67. doi: 10.1007/s44211-026-00928-8 (PMC13400482; doi:10.1007/s44211-026-00928-8)
Supplement: Supplementary file 1 — Supplementary Material 1 [file 44211_2026_928_MOESM1_ESM.docx]

Supporting Information

**Small-Molecule-Based CUT&RUN for G-Quadruplex DNA Using a Cyclic Naphthalene Diimide–Copper Complex**

Yukina SANADA, ^*1^ Satoshi FUJII,^*2^ Shigeori TAKENAKA, ^*1^ Shinobu SATO^*1†^

^*1^*Department of Applied Chemistry, Kyushu Institute of Technology, 1-1 Sensui-cho, Tobata-ku, Kitakyushu-shi, Fukuoka 804-8550, Japan*

^*2^*Department of Bioscience and Bioinformatics, Kyushu Institute of Technology, 680-4 Kawazu, Iizuka-shi, Fukuoka 820-8502, Japan*

^†^ To whom correspondence should be addressed.

E-mail: shinobu@che.kyutech.ac.jp

1. *Synthesis of cNDI-GGHE*

Structural characterization of cNDI-NMe-Glu-NH₂, a key precursor prior to GGHE conjugation, are provided below. The final product was characterized by analytical RP-HPLC and ESI-MS due to limited sample quantity and spectral complexity associated with the peptide-containing structure.

Scheme 1

Fmoc-Glu (cNDI)-OH was synthesized by the previously reported method [1] from cNDI-NMe-Glu-NH_2_ (^1^H-NMR shown in Fig. S1). R1 (0.51 g (1.3 mmol) of OxideHexafluorophosphate (Tokyo Chemical Industry Co., Ltd.) in 5 mL of DMF), R2 (140 µL (0.80 mmol) of N,N-diisopropylethylamine (Watanabe Chemical Industries, Ltd., Hiroshima, Japan) in 3 mL of DMF), 14 mL of 30% piperidine (Sigma-Aldrich, St. Louis, MO) in DMF), 14 mL of 25% Acetic Anhydride (FUJIFILM Wako Pure Chemical Co) in DMF), 5% Triisopropylsilane (Watanabe Chemical Industries, Ltd.) in TFA were prepared in advance.

0.082 g (0.036 mmol) of Fmoc-NH-SAL Resin (Watanabe Chemical Industries, Ltd.) was placed in a reaction vessel, 2 mL of DMF was added, and the mixture was left overnight. All subsequent experimental procedures were performed in a reaction vessel. To deprotect the Fmoc group, 1000 µL of 30% piperidine was added and shaken for 5 minutes (×2). The resin was then washed five times with 3 mL of DMF. 0.163 g (0.13 mmol) of Fmoc-Glu(cNDI)-OH and 500 µL of the previously prepared R1 and R2 were added to the washed resin and shaken for 1 hour. After the reaction, the resin was washed five times with 3 mL of DMF and three times with 3 mL of dichloromethane (DCM, Wako Pure Chemical Industries). To block the resin, 1000 µL of 25% Acetic Anhydride was added and shaken for 5 minutes twice. The resin was washed three times with 3 mL of DCM and five times with 3 mL of DMF twice. To deprotect the Fmoc group, 1000 µL of 30% piperidine was added and shaken for 5 minutes twice. The resin was washed five times with 3 mL of DMF. The washed resin was reacted in the same manner with 0.082 g (0.13 mmol) of Fmoc-His (Mtt)-OH (Watanabe Chemical), 0.043 g (0.14 mmol) of Fmoc-Gly-OH (Watanabe Chemical), and 0.041 g (0.14 mmol) of Fmoc-Gly-OH (Watanabe Chemical). After all reactions were completed, the resin was dried under vacuum.

After vacuum drying, 1000 µL of 30% piperidine was added to deprotect the Fmoc group, and the resin was shaken for 5 minutes twice. The resin was washed with 3 mL of DCM (×3), 3 mL of DMF (×5), 3 mL of DCM (×3), and 3 mL of methanol (×5). To cleave the resin, 1000 µL of 95% TFA was added and shaken for 3 hours, and the reaction solution was recovered. The resin was then added with 1000 µL of 95% TFA and shaken for 10 minutes (×2) and the reaction solution was recovered in the same manner. After shaking for 5 minutes with 1000 µL of methanol, the solution was recovered, and the wash solution with 1000 µL of methanol was also recovered. These recovered solutions were evaporated in an evaporator, azeotroped with methanol (×6), and vacuum dried to obtain an orange solid. The residue was added in 0.1 % TFA, the peak at R.T. 16.5 min as collected by reversed phase HPLC (Waters e2695 Separations Module, Waters 2998 Photodiode Array Detector) on Inertsil ODS-4 (inner diameter 5 mm, size 7.6 × 250 mm mm, GL Science Inc., Japan) in a gradient mode at a flow rate of 2.7 ml/min, where the concentration of acetonitrile was changed linearly to 28 % from 7.0 % in water containing 0.1% trifluoroacetic acid over 20 min. Elution was monitored by absorption at 210 and 384 nm (Fig. S1). After lyophilization, the target product was identified by ESI-MS (Fig. S2, m/z=1013.4976 (Theory for C_48_H_64_N_14_O_11_+H^+^=1013.4959)). Lyophilization left 0.021 g (0.021 mmol, 18% yield) of an orange powder.

[1] K. Ono, S. Sato, S. Takenaka, ChemistrySelect (2024) <https://doi.org/10.1002/slct.202401062>


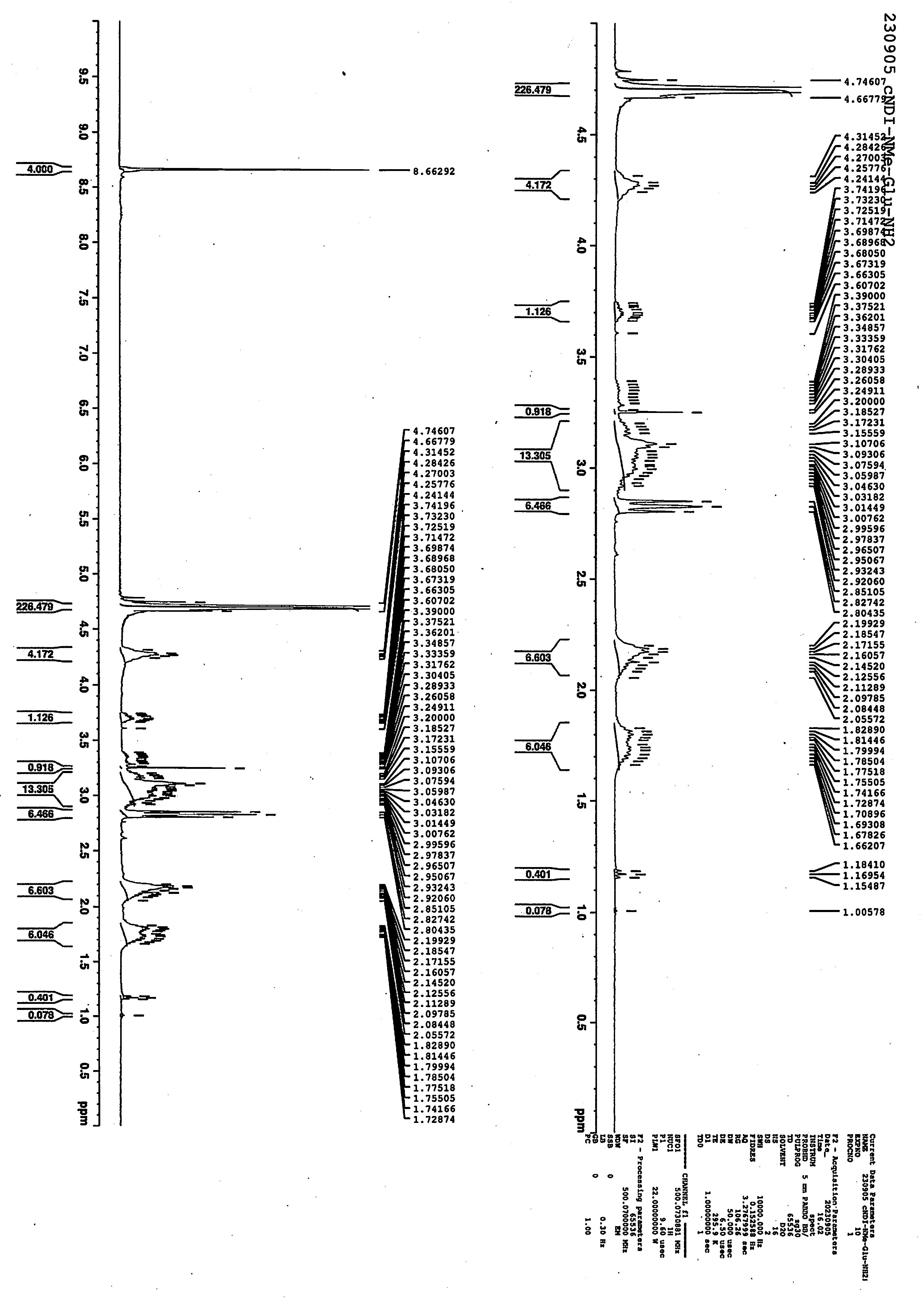


**Fig. S1**. ^1^H-NMR for cNDI-NMe-Glu-NH_2_ in D_2_O.


(C)

**Fig. S2**. Reversed HPLC of cNDI-GGHE (A) before and (B) after purification. The concentration of acetonitrile (CH_3_CN) was changed from 7% to 28% (0-20 min) and from 28% to 70% (20-30 min) in water containing 0.1% CF_3_COOH at 40 °C. (C) ESI-MS of cNDI-GGHE in H_2_O. m/z=1013.4976 (Theory for C_48_H_64_N_14_O_11_+H^+^=1013.4959).

Fig. S3. UV–Vis absorption spectral changes of cNDI-CuGGHE upon titration with each DNA; (A) c-*myc*, (B) c-*kit*, (C) TA-core, (D) G2T1, (E) ds-oligo, and (F)12-ss(–) in 50 mM AcOK-AcOH (pH 5.5), and 100 mM KCl at 25 °C.

Fig. S4. UV–Vis absorption spectral changes of cNDI-GGHE upon titration with each DNA; (A) c-*myc*, (B) c-*kit*, (C) TA-core, (D) G2T1, (E) ds-oligo, and (F)12-ss(–) in 50 mM AcOK-AcOH (pH 5.5), and 100 mM KCl at 25 °C.

**Fig. S5.** Circular dichromic spectra of 1.5 μM c-*myc* (A, F), c-*kit* (B,G), TA-core (C, H), G2T1 (D, I), or ds-oligo (E, J) in 50 mM AcOK-AcOH (pH 5.5), and 100 mM KCl at 25 °C under 0, 1.5, 3.0, 6.0, 10.5 and 15.0 μM cNDI-CuGGHE (A-E) or cNDI-GGHE (F-J) at 25˚C.00

**
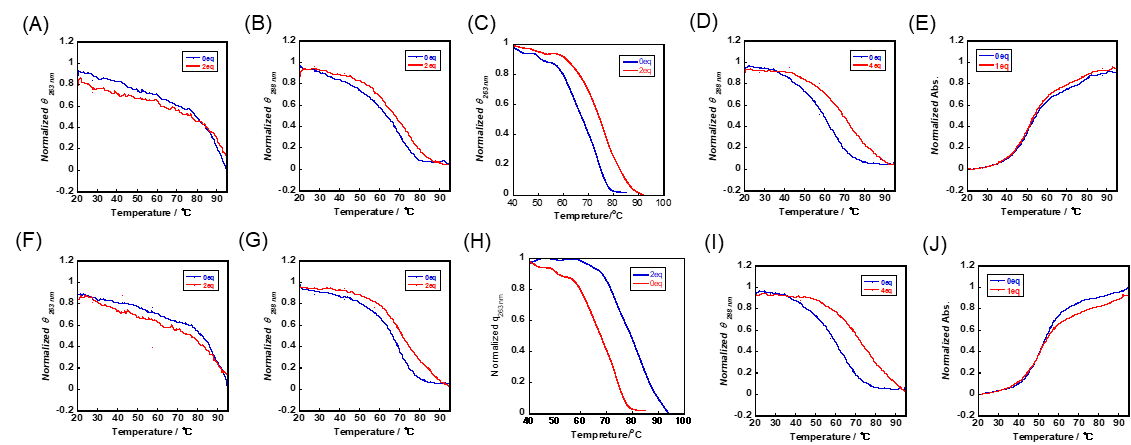
**

**Fig. S6**. Melting curves of 1.5 μM c-*myc* (A, F),TA-core (B, G), c-kit (C, H), G2T1 (D, I), or ds-oligo (E, J) with the absence (blue) or presence of cNDI-CuGGHE (A-E) or cNDI-GGHE (F-J) in 50 mM AcOK-AcOH (pH 5.5), and 100 mM KCl.

**Fig. S7.** Gel electropherogram for DNA cleavage by cNDI-CuGGHE. 1.5 μM c-kit (A), TA-core (B), G2T1 (C), ds-oligo (D) or HP-27 was treated with 1 mM NaAsc and 1 mM H_2_O_2_ at 37 °C. Lane 1, 12: 10 bp DNA ladder; Lane 2: DNA only; Lane 3: DNA + cNDI-CuGGHE; Lane 4: DNA + NaAsc + H₂O₂; Lanes 5–11: DNA + cNDI-CuGGHE + NaAsc + H₂O₂ (reaction time: 0, 2.5, 5, 10, 20, 40, 60 min).

**Fig. S8**. Reversed HPLC of 10 μM c-*myc* (A) or 10 μM TA-core (B)) before and after reaction with 1.0 µM cNDI-CuGGHE, 1.0 mM NaAsc, and 1.0 mM H_2_O_2_ for 4min at 37°C. HPLC (Waters e2695 Separations Module, Waters 2998 Photodiode Array Detector) on Mightysil RP-18 (size 4.6 × 250 mm mm, Kanto Kagaku,, Japan) in a gradient mode at a flow rate of 1.0 ml/min. The concentration of methanol was changed from 0% to 36.25% (0-30 min) and from 36.25% to 50% (30-35 min) in water containing 100 mM Hexafluoro-2-propanol and10 mM TEA at 40°C.

**Fig. S9**. Primer MAP for qPCR verification assay. FP and RP mean forward and reverse primer. Region 1 is sandwiched between FP1 and RP1, and region 2 is sandwiched between FP2 and RP2. There is a G4 site between regions 1 and 2. If the G4 site is cleaved, region 3 between FP1 and RP2 is not amplified, and only regions 1 and 2 are amplified.

Table S1. Primer list for qPCR verification assay

|  |  | Sequence |
| --- | --- | --- |
|  | FP1 | 5' -AGTACTGCTACGGAGGAGCA-3' |
| c-*myc* | RP1 | 5' -ACAGAGTAAGAGAGCCGCATG-3' |
|  | FP2 | 5' -CTCTCTCGCTAATCTCCGCC-3' |
|  | RP2 | 5' -TTCTCGAGGCAGGAGGGG-3' |
|  | FP1 | 5' -CACATCCCAGGGGTGGAAAG-3' |
| c-*kit* | RP1 | 5' -CCCTGCTCTTTCGACGTGTT-3' |
|  | FP2 | 5' -GAGAGCTGGAACGTGGACC-3' |
|  | RP2 | 5' -AAGCAGTAGGAGCAGAACGC-3' |

Forward Primer, FP/Reverse Primer, RP
